# Azygos Vein Aneurysm as a Posterior Mediastinal Mass Discovered After Minor Chest Trauma

**DOI:** 10.5812/iranjradiol.7467

**Published:** 2014-01-30

**Authors:** Gholamreza Mohajeri, Ali Hekmatnia, Hossein Ahrar, Farzane Hekmatnia, Reza Basiratnia

**Affiliations:** 1 Department of Surgery, Al-Zahra Hospital, Isfahan University of Medical Sciences, Isfahan, Iran; 2 Department of Radiology, Image Processing and Signal Research Center, Al-Zahra Hospital, Isfahan University of Medical Sciences, Isfahan, Iran; 3 London University of Medical Sciences, London, UK

**Keywords:** Azygos Vein, Aneurysm, Mediastinum, Tomography, X-Ray Computed, Magnetic Resonance Angiography

## Abstract

Azygos vein aneurysm is a rare cause of a posterior or paratracheal mediastinal mass. Trauma or conditions causing elevated ﬂow or pressure in the azygos system, such as cardiac failure or cirrhosis of the liver are secondary causes of aneurysm of the azygos vein. We report a case of asymptomatic saccular aneurysm of the azygos vein in a 45-year-old man with blunt minor chest trauma. The azygos vein aneurysm was managed by conservative treatment.

## 1. Introduction

Azygos vein aneurysm is a rare cause of a posterior or paratracheal mediastinal mass (1, 2). Most reported cases are secondary to trauma or conditions such as portal hypertension or heart failure (3, 4). Treatment of the azygos vein aneurysm is conservative and excision is recommended in complications such as rupture, obstruction or embolic events and in suspicious diagnosis (2, 5).

## 2. Case Presentation

**Figure 1.**
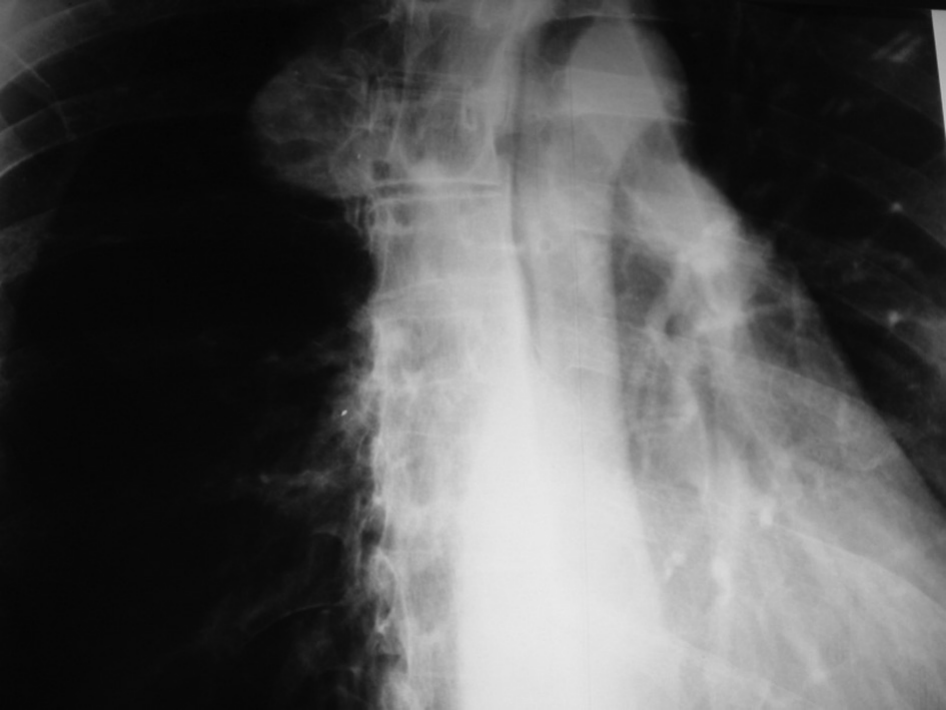
PA CXR showing a right sided round soft tissue mass lesion probably in the right posterior mediastinum

A 45-year-old man was referred to the emergency department due to chest pain following fist blow to the anterior chest wall during sport practice. No changes in vital signs or laceration were detected. In his medical history, there was no evidence of hospitalization, surgery or clinical conditions. A PA chest X-ray showed a right sided large spherical shadow with clear-cut margins probably in the posterior mediastinum (Figure 1). 

**Figure 2.**
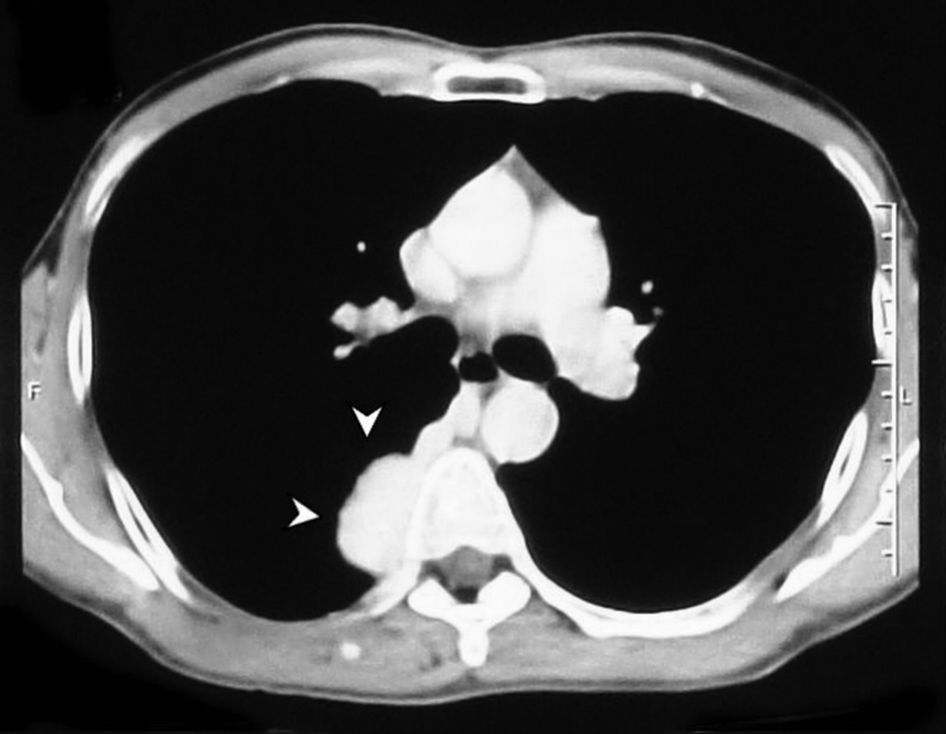
Axial CT scan at the level of the carina shows a lobulated enhancing soft tissue lesion in the right posterior mediastinum

Dynamic CT confirmed a lobulated tubular right paravertebral mass approximately 38×28 mm^2^ in size, with marked enhancement, without a bony lesion; all pointing to a vascular structure. The mass was located in the position of the azygos vein (Figure 2). 

**Figure 3.**
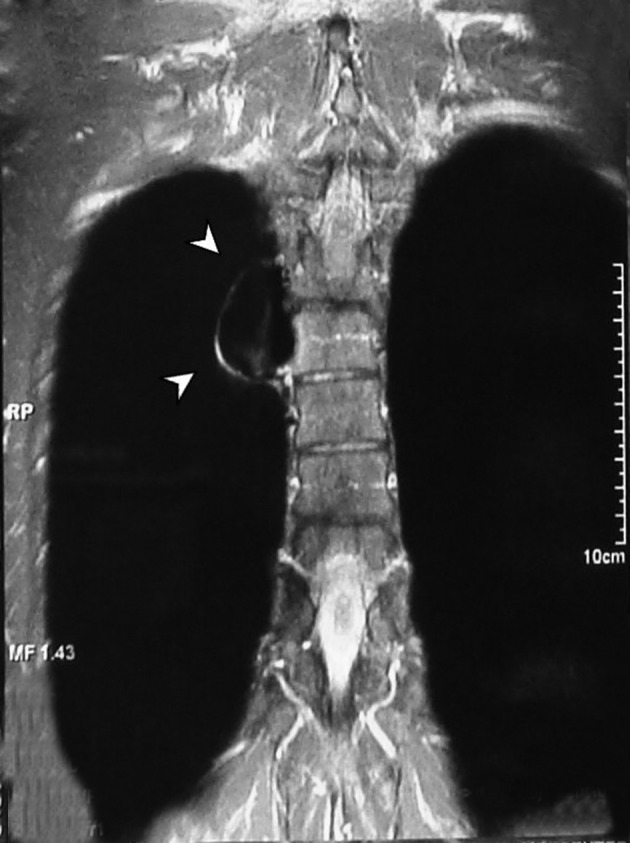
MRI of the thoracic region showing signal void tubular structure in the right paravertebral space

**Figure 4.**
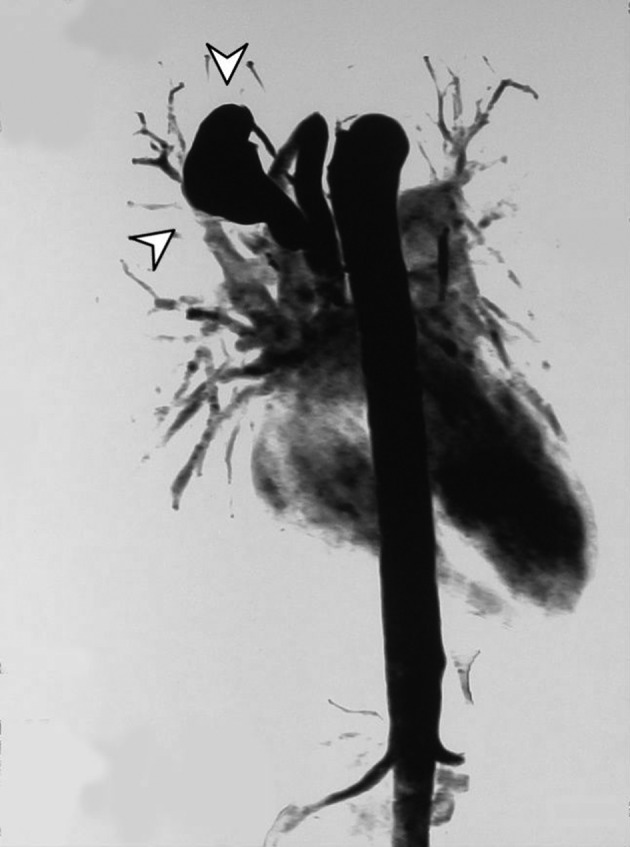
Contrast enhanced MRA study of the thoracic aorta demonstrating a saccular enhancing vascular lesion with a wide neck connecting to the azygos vein on the right side

A tubular lesion in the right posterior mediastinum was seen with signal void appearance in all pulse sequences of the MRI study (Figure 3). Magnetic resonance angiography (MRA) showed an enhancing saccular right paravertebral vascular lesion draining to the azygos vein through a wide neck in the thoracic region. With regards to the abovementioned findings, the impression was aneurysmal dilatation of the azygos vein (Figure 4). 

**Figure 5.**
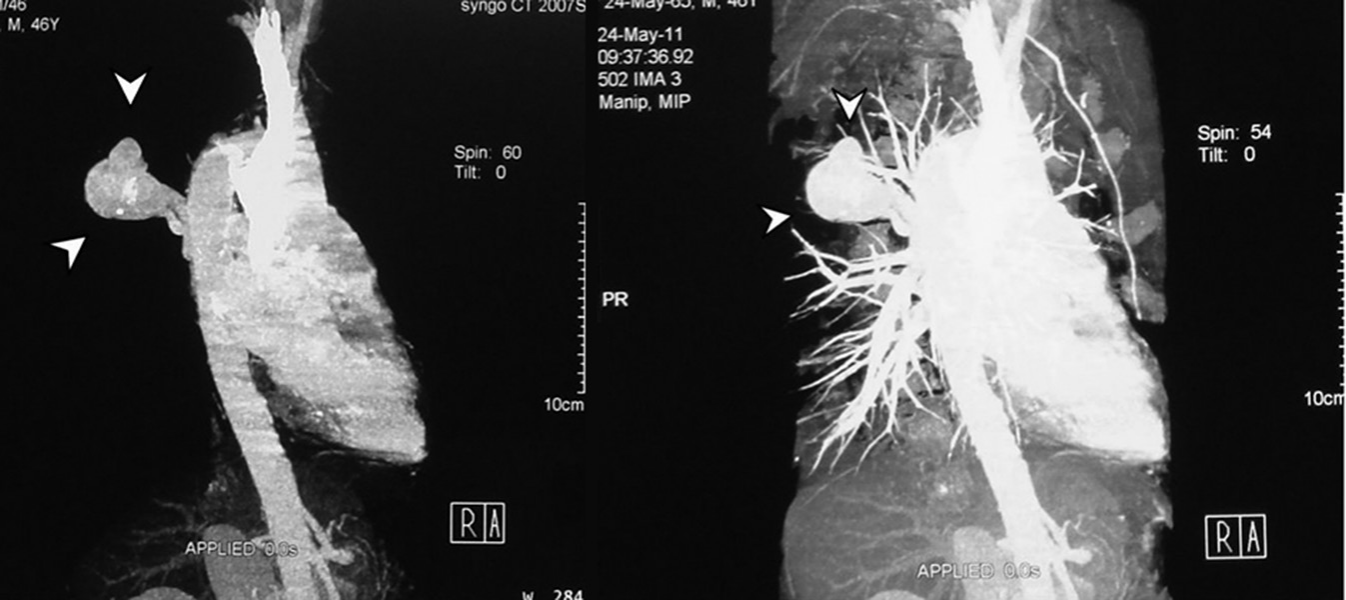
CT angiography after 3 months. No growth or thrombosis was detected.

As far as detected in imaging studies, there is no other anomaly or pathologic finding in vascular territories such as IVC or SVC. Due to the absence of symptoms and thrombosis in the aneurysmal vein, conservative treatment was chosen. In CT angiography after 3 months, no growth or thrombosis was detected (Figure 5). The patient has been asymptomatic over the past year. 

## 3. Discussion

Aneurysm of the azygos vein is exceedingly rare and few cases have been described in the literature. The first one was reported in 1981 by Barraine et al. (6). The importance of the aneurysm of the azygos vein might be due to its appearance as an incidental finding in imaging studies. It is considered in the differential diagnosis of a mass in the area of the right tracheobronchial angle or of an enlargement of the right upper or posterior mediastinum. Dynamic computed tomography and MRI are very important in diagnosing this abnormality and confirming its anatomical relationships (1, 3, 5).

There are three main causes of azygos vein aneurysm. Most reported cases are secondary to trauma including blunt injury or catheter insertion into the azygos vein.

The other one is azygos vein dilatation caused by pressure and volume load secondary to congestive heart failure, obstruction of the superior or inferior vena cava, congenital anomalies of the inferior vena cava like agenesis and thrombosis, portal hypertension, pregnancy and pulmonary sequestration with azygos drainage. This type of aneurysm is usually fusiform (1, 2, 4). Some believe a congenital origin for azygos aneurysm as the third reason. Abnormal development of forming vessels of the azygos vein, right supracardinal and the proximal portion of the posterior cardinal vein can be suggested as a cause of idiopathic azygos vein aneurysm (4, 5). It usually presents as an asymptomatic mass (4, 5). Symptoms depend on the size of the aneurysm. When the aneurysm is enlarged, the mass effect on the esophagus, bronchus and superior vena vena cava are successively prospected. Pulmonary embolism as a result of thrombus formation within the aneurysm can be the potential complication (1).

On chest X-ray, a well-defined mass is revealed in the right tracheobronchial angle that has a variable size during inspiration or the Valsalva maneuver. Depending on the size of the neck of the aneurysm, there is only slight enhancement of the mass in the early phase, but homogeneous enhancement in the late phase on dynamic computed tomographic images (1, 3, 5). MR images are variable based on the neck width of the aneurysm; from low signal intensity in both T1 and T2 weighted images in a narrow neck to mixed signal density in wide neck aneurysms. The width of the aneurysmal neck affects the amount of blood flow within the aneurysm (5).

 In our case, the mentioned aneurysmal dilatation had a wide neck causing high blood flow in it. Therefore, signal void appearance in all pulse sequences of MRI study could be expected (Figure 3). Treatment of the aneurysm of the azygos vein is controversial. Mostly, the literature has limited surgical intervention to complications such as rupture, obstruction or embolic events (2, 4).

## References

[1] Braun P, Guilabert JP, Olaso LT (2004). Aneurysm of the azygos arch.. Eur J Radiol Extra..

[2] Lee DH, Keum DY, Park CK, Kim JB, Rho BH (2011). Azygos Vein Aneurysm - A Case for Elective Resection by Video-assisted Thoracic Surgery.. Korean J Thorac Cardiovasc Surg..

[3] Watanabe A, Kusajima K, Aisaka N, Sugawara H, Tsunematsu K (1998). Idiopathic Saccular Azygos Vein Aneurysm.. Ann Thora Surg..

[4] Gnanamuthu BR, Tharion J (2008). Azygos vein aneurysm--a case for elective resection.. Heart Lung Circ..

[5] Revilla Y, Martı A, Nez E, Benito J (2003). Azygos vein diverticulum, a rare case of a mediastinal mass lesion.. Eur J Radiol Extra..

[6] Barraine R, Gasquet C, Cabrol C, Tournoux B, Bordage JP, Bonneau A (1981). Les anevrysmes de la veine azygos.. Arch Mal Coeur..

